# Chronic Kidney Disease and Arterial Stiffness: A Two-Way Path

**DOI:** 10.3389/fmed.2021.765924

**Published:** 2021-11-23

**Authors:** Felipe Inserra, Pedro Forcada, Agustina Castellaro, Carlos Castellaro

**Affiliations:** ^1^Advisor of Academic Vice-Rectory Department, Maimonides University, Buenos Aires, Argentina; ^2^Master Vascular Mechanics and Arterial Hypertension, Postgraduate Department, Hypertension, Austral University, Buenos Aires, Argentina; ^3^Non-Invasive Vascular Labs, CardioArenales and Diagnóstico Integral Médico (DIM) Prevención Cardiovascular, Buenos Aires, Argentina; ^4^Pediatric Medicine of Prof. Dr. Juan P Garrahan Hospital, Buenos Aires, Argentina; ^5^Department of Nephrology, Centro de Educación Médica e Investigaciones Clínicas Norberto Quirno (CEMIC) Hospital, Buenos Aires, Argentina

**Keywords:** pulse wave velocity (PWV), calcification, CKD progression, pulsatility, cardiovascular events

## Abstract

The kidney-heart relationship has raised interest for the medical population since its vast and complex interaction significantly impacts health. Chronic kidney disease (CKD) generates vascular structure and function changes, with significant hemodynamic effects. The early arterial stiffening in CKD patients is a consequence of the interaction between oxidative stress and chronic vascular inflammation, leading to an accelerated deterioration of left ventricular function and alteration in tissue perfusion. CKD amplifies the inflammatory cascade's activation and is responsible for altering the endothelium function, increasing the vascular tone, wall thickening, and favors calcium deposits in the arterial wall. Simultaneously, the autonomic imbalance, and alteration in other hormonal systems, also favor the overactivation of inflammatory and fibrotic mediators. Thus, hormonal disarrangement also contributes to structural and functional lesions throughout the arterial wall. On the other hand, a rise in arterial stiffening and volume overload generates high left ventricular afterload. It increases the left ventricular burden with consequent myocardial remodeling, development of left ventricular hypertrophy and, in turn, heart failure. It is noteworthy that reduction in glomerular mass of renal diseases generates a compensatory glomerular filtration overdriven associated with large-arteries stiffness and high cardiovascular events. Furthermore, we consider that the consequent alterations of the arterial system's mechanical properties are crucial for altering tissue perfusion, mainly in low resistance. Thus, increasing the knowledge of these processes may help the reader to integrate them from a pathophysiological perspective, providing a comprehensive idea of this two-way path between arterial stiffness and renal dysfunction and their impact at the cardiovascular level.

## Introduction: The Role of Vascular Injury in the Kidney and Cardiac Disease Interaction

A robust functional relationship exists between the kidneys and the cardiovascular system, and this vast and proven interaction has acquired progressive notoriety.

The evidence from population studies clearly shows that renal and cardiac diseases are strongly associated (1, 2). This evidence includes the different clinical situations where structural lesions (tissue, cellular, and subcellular) develop simultaneously with both organs' progressive functional deterioration (3–5).

Of particular interest is the relationship between the heart and kidneys when both are functionally insufficient. This interplay is known as the cardiorenal syndrome, and the most relevant are chronic types 2 and 4 of the original description by Ronco et al. (6). This interaction is also known as “the cardiorenal link” (7).

The pathophysiological and histopathological concepts involved, including the graphics and figures, are designed to understand better the kidney and the heart's role as principals organs involved.

However, these conceptual proposals do not define the precise role or sequence of the macro and microcirculation structures in this process. Therefore, we tried to analyze the process of vascular injury in CKD (8–10), giving the central role it plays and its interaction with different tissue injuries leading to the cardiorenal syndrome's progression. The leading role of the circulation (macro and micro) and its interaction with the kidneys different pathophysiologic hypotheses on intrarenal hemodynamics changes that they generate.

### General Concepts of Vascular Behavior

The essential objective of blood traveling through the arterial system is the perfusion and oxygenation of peripheral tissues. Under normal conditions, the cardiac pump discharges the systolic volume (SV) of blood received by the large-caliber elastic arteries, mainly the aorta. However, ~50% of the previously mentioned SV is dampened due to the compliance of its walls.

The remaining 50% of the SV continues its way to the peripheral arteries. Once the aorta returns to its initial caliber in diastole, its elastic capacity, if it is healthy, sends the remaining volume forward, transforming the arterial flow from pulsatile to continuous in the peripheral circulation. This fact is known as the “Windkessel Phenomena” in comparison to the old fire extinguishing pump.

At the distal level, the arteries have a structural component mainly integrated by smooth muscle cells coupled “in series” with collagen fibers, all influenced by neural and hormonal factors. This massive parallel resistance system is responsible for peripheral vascular resistance and the dissipation of at least two-thirds of the cardiovascular system's pulsatile energy. This fact allows arterioles to adapt to different situations, organs, and pathophysiological circumstances. However, this regulable system loses efficacy because of aging (normal or accelerated) and other conditions—such as high blood pressure, diabetes, and CKD—that cause a decrease in arterial compliance due to the loss of the vessel's elastic components. The half-life of elastin, the main factor responsible for the aorta's elasticity, is measurable in years. Continuous and intermittent distension of the aorta with each heartbeat and during the lifespan causes fatigue and fracture of the elastin fiber, leading to increasing stiffening of the aorta's wall (11). In this scenario, the accumulation of different collagen types that are stiffer than the initial one, and other substances like Advanced Glycation End Products (AGEs) occur, conducting the loss of compliance of the elastic arteries (11, 12). When this loss of arterial compliance, or its opposite, an increase in arterial stiffness, evolve faster than expected by normal aging, we are in the presence of “early vascular aging” (EVA). Certain metabolic disorders and diseases cause this EVA phenomenon to accelerate and appear in earlier stages or with greater severity. Kidney disease is a frequent cause and one of the most representative examples of this phenomenon.

## One Way: From Chronic Kidney Disease to Vascular Injury

During the development and progression of CKD, the aortic compliance decreases, reducing SV's buffering capacity, resulting in an exaggerated increase of the systolic pressure (SP) and a drop of the diastolic pressure (DP).

The pulse pressure (PP) or pulsatility is the difference between SP and DP in mmHg. Hence, it is an easy parameter to be observed during the medical examination when measuring blood pressure.

A stiff aorta produces the loss of its properties as a second pump, or “second heart” (a consequence of the lack of elastic recoil of a distensible aorta), with the consequent drop in the diastolic vascular flow and pressure. Aortic stiffness also increases the pulsatility in the peripheral vessels and their irrigated tissues. Due to these conditions, the SV is no longer buffered and continues toward the periphery.

On the other hand, arterial wall stiffening increases the pulse wave velocity (PWV), the speed at which the pulse wave travels through the large arteries' wall, and is currently considered the gold standard for arterial stiffness measurement. It is also an independent prognostic marker of CVE (13, 14). The increase in SP results in increased heart afterload, augmented work of the left ventricle (LF), and increased oxygen consumption by the myocardium. Over time, the persistent imbalance favors the development of left ventricular hypertrophy (LVH) and heart failure (HF).

**Figure 1:**
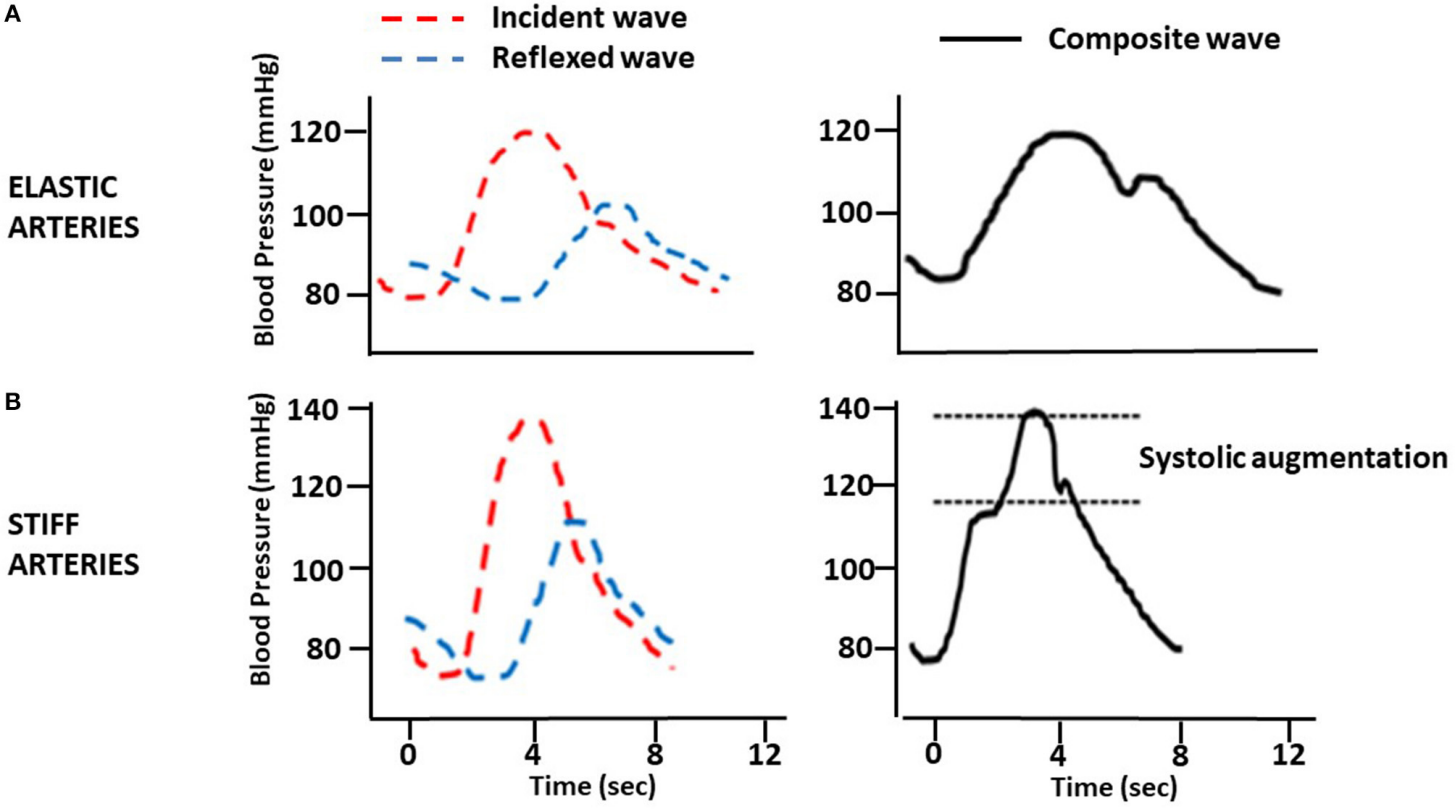
Changes in pulse wave by arterial stiffness. **(A)** Elastic arteries. **(B)** Stiff arteries.

Another physiological phenomenon is related to the highest speed of the PWV. Under physiological conditions, the incident wave, generated from the systolic discharge, propagates through the arterial system to the reflection points. These sites are the arterial bifurcations or regions of the most significant change in the arterial wall's viscoelastic components. A reflected wave returns in the opposite direction during late systole and adds to the new incident wave, giving rise to the augmentation wave phenomenon, measured as “augmentation index” (Aix) (Figure 1A).

In situations of increased arterial stiffness, as occurs in CKD, through mechanisms described in this review, the wave's reflection occurs earlier and arrives prematurely, worsening the Aix, considered an marker of arterial stiffness (Figure 1B). Therefore, an additional increase in central aortic pressure (CAP) contributes to increasing cardiac work and oxygen consumption, both of which provoke further activation on the pathways and mechanisms leading to LVH and HF.

A direct relationship stands between renal function impairment with CAP and Aix. Thus, the more significant the CAP and Aix are, the more influential they are on renal function deterioration and death, as was demonstrated by Towsend et al. in the CRIC study (15, 16). Recent data confirmed that Aix was independently associated with mortality in CKD patients after adjusting for additional confounders, including inflammation (17).

In other words, the increase in arterial stiffness -represented by the rise in PWV- is usually associated with an increase in the SP with consequent LV overload. In addition, the increase in PWV and vascular stiffness speeds up the pulse wave's reflection, generating an increase in the aortic augmentation that further increases the CAP, and therefore, left ventricle contractile effort.

So, EVA is a consequence of cardiovascular risk factors (CVRF), amplified by CKD, and this cluster of factors lead to premature cardiovascular events (CVE) (18–20), as well as accelerate the damage of various tissues and their functions, including a faster decline of kidney function.

### Association Between Inflammation and Arterial Stiffness

**Figure 2:**
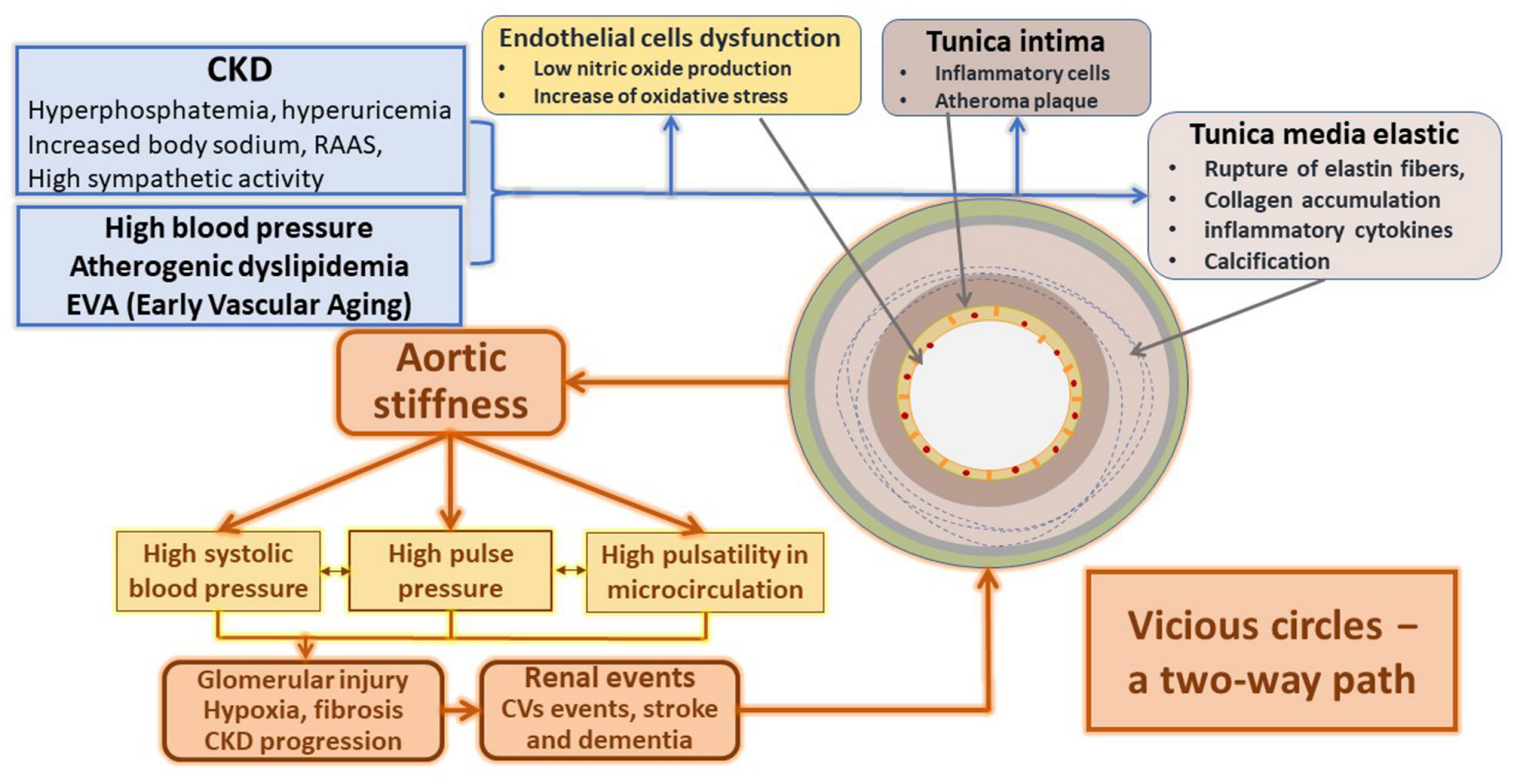
Main mechanisms responsible for the structural and functional changes of the arteries in CKD.

The different tissue lesions associated with CVRF are also related to the activation of diverse inflammatory ways. Initially, they act as a protective response of the organism to control the cause, but finally, they turn into a disease. The inflammatory response to stressors acts on the endothelium and vascular smooth muscle; therefore, serum and tissue inflammatory markers are tools that help to predict cardiovascular disease (CVD) (21–23). Figure 2 summarizes the primary inflammatory mechanism associated with vascular stiffness in several clinical situations, particularly CKD.

The association between chronic inflammation and arterial wall disease is complicated and multifactorial (24). Initially, the circulation of inflammatory mediators favors leukocytes' migration into the arterial wall (25). Then, macrophages activation by different factors, including metabolic and electrolytic disturbances associated with catecholamines, renin-angiotensin-aldosterone system (RAAS), and endothelin disarrangement associated with cytokines and reactive oxygen species (ROS), amplify the inflammatory reaction.

The subsequent transformation of these macrophages within the arterial wall into foamy cells predisposes to their necrosis; when the necrotic nucleus appears in the plaques, the amplification of the inflammatory stimulus favors the progression of already advanced vascular lesions (26).

This inflammatory cascade also alters the endothelium's function that interacts and conditions the remodeling of the tunica media and changes of the artery's mechanical properties (23). Endothelial cells decrease the usual production of nitric oxide (NO) and increase endothelin (E1), favoring arterial stiffness. In turn, arterial stiffness subsequently alters the endothelium, thus generating a vicious circle (27, 28).

Simultaneously, the increase in arterial stiffness and the dysfunctional endothelium activate adhesion molecules like MCP-1 and cytokines favoring thrombotic events (21, 29, 30). Dendritic cells and T-lymphocytes play an essential role in synthesizing pro-atherogenic cytokines (IL-2, IL-18, and IFN-gamma), responsible for the installation and progression of atherosclerotic plaques (31).

Vascular inflammation enhanced by CKD promotes the vessel's stiffening by stimulating fibrosis and proliferation of the vascular smooth muscle cells (VSMC) (23).

### Role of CKD in the Inflammatory State and Vascular Injury

CKD, defined as a structural and functional alteration of the kidney for more than 3 months (32). CKD is a low-grade chronic inflammatory state associated with a significant increase in morbidity and mortality (33). CKD and a set of factors—chronic acidosis, recurrent infections, and altered microbiota—generate increased cytokine production, oxidative stress, and inflammation.

CKD resembles an experimental oxidative stress model which produces severe alterations in many cells (nuclear and mitochondrial DNA deletion, telomeric shortening), tissues, serum, and urinary markers. Oxidative stress is an initial and central contributor to endothelial dysfunction and the inflammatory process, conducting atherosclerotic vascular injury, premature aging, and CVD (34). A decrease in anti-aging defenses (like Klotho and Fetuin-A activity) increases pro-aging mediators such as angiotensin II, aldosterone, and phosphate, generating a clear discrepancy between chronological and vascular biological age (35, 36).

Inflammation that accompanies kidney disease seems to play a significant role in telomeric shortening and mitochondrial dysfunction (37). Additionally, Galvan et al. have shown a low number of mitochondria, also dysfunctional, in most of the tissues of patients with CKD, representing a primary metabolic-energetic alteration present in these patients from very early stages (38). It is an essential component of the disease and the primary source of increased reactive oxygen species production. This inflammatory state also decreases the body's resistance to external stressors, thus conditioning a state of increased vulnerability (33).

At the same time, the kidney itself is vulnerable to this inflammatory process. The kidneys are intensely and heterogeneously vascularized and regulated by hormones and vasoactive molecules (like RAAS, prostaglandins, endothelin, NO, and others) (39, 40). Systemic inflammation favors the intrarenal inflammatory cascade associated with tubular and glomerular injury and, therefore, generation and progression of CKD.

Systemic inflammation eases the development of renal injury and is co-responsible for the high morbidity and mortality of these patients and the development of an accelerated aging phenotype.

In CKD, calcification of the middle layer of the arteries is a part of the accelerated EVA process. Therefore, the extent of vascular calcium vascular deposits is related to vascular estimated age. That is why in CKD, the age of the vasculature is practically always older than the chronological age, at least partially, due to an early and persistent inflammatory process.

Unlike what happens in individuals with preserved kidney function, those with CKD have a process of accelerated cellular and vascular senescence, tissue aging, persistent inflammation, loss of muscle mass, osteoporosis, and early general fragility.

In other words, EVA in patients with CKD is part of the price paid for the enormous allostatic load, a consequence of multiple physical and inflammatory stressors associated with CKD.

### The Partial Reversion of Vascular Changes by Renal Transplantation

It is to point out that recovering kidney function with a kidney transplant (Tx) decreases the mortality rate by 50% compared to the same patient population submitted to dialysis treatment. Hence, it raises whether a kidney transplant may reduce the described vascular changes suffered along with CKD.

Few studies have shown after transplant regression of the large arteries remodeling; however, there is evidence about the decrease of the PWV after Tx, which increases the patient and the graft survival rate (41, 42).

The French Group Karras published the data of 161 consecutive Tx patients. The outcome was the arterial parameters, measured at 3 and 12 months after kidney transplant. The results showed that mean PWV decreases from 10.8 m/s in the 3rd month to 10.1 m/s after 12 months (*p* < 0.001). After the multivariate analysis, the patients who received Tx living donor allograft had a more significant decrease of the PWV (*p* < 0.001). Furthermore, the patients who received deceased allograft with standard donors had better vascular performance than those who received allograft from donors with expanded criteria (older donors and pre-establish cardiovascular disease). An interesting point to highlight in this study is the non-relationship between vascular function improvement and glomerular filtration level (43). The progression of arterial stiffness after 12 months of kidney Tx was also studied in 28 patients, as a control group was studied, 23 hemodialysis patients. The decrease in the PWV, measured with SphygmoCor, in Tx patients was higher than patients under hemodialysis treatment (*p* < 0.0001) (44).

Korogiannou et al. recently confirmed the relevance of PWV in the prognosis of Tx patients been a strong predictor for cardiovascular events, renal events, and mortality in these individuals (45).

Although scientific facts are clear enough, there are still two significant aspects to consider. First, there are no clinical trials about the impact of therapeutic interventions on arterial stiffness and its consequence on the kidney Tx population. Also, mechanistic studies are required to identify the best ways to address arterial stiffness in Tx patients.

## Uremic Toxins as Vascular Toxins

**Hyperphosphatemia and its consequences**: During CKD, an imbalance between the inhibitors and inducers of vascular calcification occurs (46).

The decrease in renal phosphate excretion increases serum levels and promotes the calcification process by activating the Toll-like receptor four and NF-Kappa B in VSMC (47). Also, in the context of hyperphosphatemia, VSMC changes its phenotype to osteoblastic-like cells *via* the expression of ossifying genes (48). Likewise, phosphates also produce mitochondrial dysfunction, with increased reactive oxygen species production, activation of pro-inflammatory molecules, and increased tumor necrosis factor (TNF).

CKD also alters hormonal processes that regulate phosphate levels (Intestinal absorption, renal excretion by remaining nephrons, bone metabolism modulated by vitamin D, fetuin-A, Klotho, and fibroblast growth factor 23 (FGF-23) (49). Calcium deposits concentrated in the tunica media and the vascular wall's subendothelium are an essential part of the problem. A detailed description of the facts exceeds this manuscript's objectives.

Uric acid increases in CKD due to the decrease excretion by the failing kidney. This mentioned uric acid elevation decreases endothelial Nitric Oxide Synthase (eNOS) activity, reducing the production of NO, the proliferation of VSMC (50, 51), the expression of COX-2, and the increase in the production of angiotensin II, contributing to arterial stiffness.

**Advanced glycation ends products (AGEs)**: AGEs accumulate in CKD progressively as their production increases and elimination decreases. Thus, significant accumulation may occur even in non-diabetic patients. AGEs, among other things, affect the activity of eNOS (52), favor the phenotypic change of the VSMC, and the “cross-linking” of collagen (the changes of its composition make the arterial wall less compliant). They also activate NF Kappa B, favoring the activation of the vascular inflammatory cascade and structural stiffening.

The same happens with the increase in asymmetric dimethylarginine (ADMA) resulting from increased production and less excretion, contributing to a significant reduction of eNOS and consequent endothelial dysfunction. Increased ADMA also causes sympathetic stimulation, inflammation, vascular stiffness, and LVH (53–56).

**Increment of endothelin-1 (E-1) level**: E-1 is a potent vasoconstrictor implicated in cardiovascular and renal diseases. An increase of E-1 has the same origin as the increase of ADMA and uric acid. It acts on receptors with antagonistic function (ET_A_ and ET_B_ receptors), predominantly the action of ETA receptor, which is responsible for: endothelial dysfunction, increased vascular tone, inflammation, and calcification (57).

Renal ET-1 production increases associated with CKD progression, and a cluster of conditions frequently present in these patients, such as diabetes, insulin resistance, obesity, immune system activation, atherogenic dyslipidemia, nitric oxide deficiency, and oxidative stress (58).

### Vascular Inflammation in Dialysis Patients

The dialysis procedure generates additional inflammation that adds to those already described and known in CKD. All inflammatory cytokines are markedly high in dialysis patients (IL-1, IL-6, IL-23, and TNF alfa), as well as high-sensitivity C-reactive protein and fibrinogen. Albumin, as an acute-phase reactant, is decreased (59).

Frequent infections, thrombotic events, dialysate quality, and its impurities are also powerful inflammatory stimuli. Uremia increases intestinal permeability to bacteria, and this, in turn, generates more inflammation. The diets indicated in these patients (Low in potassium and phosphorous) alter the microbiota, causing dysbiosis with significant inflammatory effects. Dialysis patients usually have markedly high inflammatory markers associated with severe arterial injuries that, in turn, progress faster (60).

## Arterial Stiffness and the Barorreflex Function

The baroreflex system regulates blood pressure changes, and their proper function enables immediate regulation of it at practically constant values. Its appropriate process depends mainly on arterial compliance. Vascular lesions are widespread at the carotid and aortic levels and affect the baroreflex system's receptors. Receptors activation requires good arterial compliance; potassium channels and sodium-potassium pump regulated by a paracrine function, mainly by prostacyclin.

The combination of endothelial dysfunction and arterial stiffness produces a decrease in prostacyclin production and less arterial compliance. Consequently, less baroreflex activation occurs, causing more significant variability in arterial pressure (i.e., greater blood pressure drop when standing up). Simultaneously, there is a greater renal afference toward the central nervous system in CKD. An increase in the sympathetic activity, a further increase in the vascular tone favors LVH, CVD, and increased mortality (61). The high prevalence and severity of baroreflex dysfunction in CKD patients were recently reviewed, and how afferent and efferent pathways between kidney and brain may deteriorate its function (62, 63).

### From Arterial Stiffness to Myocardial Dysfunction

Arterial stiffness and CKD volume overload generate myocardial dysfunction directly proportional to the degree of renal failure (64).

This myocardial involvement resulting from increased pre and afterload is also associated with cardiac interstitial fibrosis, alteration of the cardiac microcirculation, and myocardial neuro-humoral activation (65).

The most common and earlier stage of ventricular dysfunction in CKD patients is diastolic failure. It is also known as HF with preserved ventricular function, the usual echocardiographic finding in CKD patients (66).

These patients frequently have other risk factors for diastolic failures, such as type 2 diabetes, high blood pressure, coronary heart disease, and accelerated aging, all contributing to maintaining and worsening diastolic dysfunction.

The structural changes of the heart in CKD include myocardial hypertrophy and thickening of the intramural arteries (67) as an adaptative response to changes in volume and pressure. Finally, what was initially an adaptative response, leads to myocardial fibrosis due to all the metabolic and neuro-humoral disorders previously described.

Other CKD alterations that further aggravate myocardial dysfunction are the over activation of systemic and intrarenal RAAS, the anemia that characterizes patients with CKD, vitamin D deficiency, and other mechanisms recently described, such as activation of mTOR, G-protein activation, and T-cell activation (7); all of them may influence cardiac structure and function. In addition, the synergy of all these factors activates apoptosis and autophagy pathways, which increase the production of extracellular matrix in the myocardium, and lead to decreased left ventricular compliance since fibrotic tissue predominates over the cardiac muscle.

The clinical consequence of all these processes is a shift to the left of the pressure-volume curve. Small changes in volume significantly increase intraventricular pressure due to cardiac compliance loss and can cause pulmonary congestion. Conversely, slight volume depletion can impair left ventricular filling and cause a decrease in systolic volume, leading to hypotension and hemodynamic instability (66).

In other words, patients with CKD have a low range of tolerance to volume changes, extrapolated to body weight, to go from volume overload to hypotension, generating an increase in hospitalizations for decompensated heart failure. In addition, in some patients, particularly the young, functional damage to the left ventricle due to volume overload may not be evident, but it will deteriorate cardiac function if overhydration persists (68).

In addition to the CKD-dependent changes in vascular structure and function already described, we must add those that depend on the diseases frequently associated. Those most common are coronary artery disease, vascular injuries, and remodeling that depends on high blood pressure, atherogenic dyslipidemia, diabetes, and their associated metabolic disorders, together with accelerated vascular aging to these pathologies.

The detailed description of these processes exceeds the objective of this publication. Several reviews (69–75) are available that deal extensively with these factors' influence on vascular changes and cardiac disease. Figure 2 shows a comprehensive synthesis integrating the main mechanisms that generate the systemic vasculature alterations in CKD.

## The Other Way: From Vascular Injury to Chronic Kidney Disease

Under a healthy vascular condition, the large arteries' elasticity moderates the cardiac pulse pressure, dampening its intensity, achieving a continuous flow of blood, in most tissues, with a low variation in arterial pressure between systole and diastole. That means a low pulse pressure. This low arterial pulsatility enters from the macrocirculation to the microcirculation, where it receives additional attenuation in the arterioles and results in microvasculature protection. However, in response to aging (76), obesity, diabetes mellitus, and mainly CKD, an increased arterial stiffening reduce the central arteries' buffering capacity, generating high pulsatile stress at the microvasculature level.

As a result, the high pulsatility introduced into the organs; of particular interest are those tissues with high viscous components such as the brain and the kidneys; both are characterized by low resistance and high flow systems, thus receiving a high volume of blood (77, 78). The main consequences of these events will be functional deterioration with the development or acceleration of cognitive disorders and renal function impairment (79). Considering that high pulsatility causes damage to the microvasculature, strategies to reduce it could slow the progression of kidney disease and associated events (80).

Several renal diseases that reduce renal mass generate an adaptive high filtration rate by a single nephron. This process also happens in diabetes, obesity, hypertension, and aging. These clinical conditions are of great interest due to their high frequency.

Glomerular hyperfiltration (GHF) or single nephron hyperfiltration is an increased glomerular filtration rate above normal values due to increased filtration per nephron unit. There is robust evidence that GHF is a risk factor for the progression of chronic kidney disease and CV events, independently of albuminuria and other factors. Remarkably, in patients with GHF, an increase in PP was proved, measured during 24-h, suggesting an association with increased large-artery stiffness and vascular damage, leading to increased CV events (81, 82).

GHF can occur in individuals with high, normal, and low GFR, as happens in most CKD patients moderate/severe stages (83). In CKD patients with low GFR, the diagnosis of GHF in the clinical practice is very challenging, but the association exists in most patients. We had previously mentioned the usual association of GHF with arterial stiffness and high PP and pulsatility.

Pieces of evidence generated more than two decades ago confirmed that pulsatile hypertension-induced glomerular distention produces changes at a cellular level and the extracellular matrix's metabolism. The changes due to mesangial cell mechanical strain occur in the remnant kidney and play an essential pathogenetic role in renal lesions. These changes were initially described in experimental diabetes and renal failure by glomerular mass reduction as the experimental model of 5/6 nephrectomy (84, 85).

Of interest, renal blood flow increases together with dilation of the afferent arteriole in the enlarged glomeruli of both models, animals with diabetes, and subtotal nephrectomies. Therefore, systemic blood pressure is transmitted into the glomerulus without the usual regulation, generating a high pulsatile stretching in the glomerular and surrounding structures. This persistent pulsatile stretch, in turn, changes the phenotypes of mesangial cells that increase the production of different cytokines producing the recruitment of cells leading to inflammation and kidney fibrosis (86, 87).

More recently, using Doppler devices, it is possible to evaluate renal microvascular pulsatility. The pulsatility index derived from pulsed-wave Doppler measurements correlates with effective renal plasma flow in CKD patients and predicts renal disease progression (88, 89).

These pathophysiological mechanisms described before are in line with consistent epidemiological results that show the association between arterial stiffness, microcirculation pulsatility, and the incidence and progression of renal diseases, as well as hard renal endpoints (90, 91).

Reducing or controlling GHF and restoring to normal the disturbed glomerular hemodynamics has been the most crucial strategy for glomerular protection and to slow the progression of chronic kidney disease. Paradigmatic drugs in kidney protection, such as those that block the renin-angiotensin system (RAS inhibitors), or the new ones such as sodium-glucose cotransport type 2 inhibitors (iSGLT2), and the GLP-1 receptor agonists (GLP-1), produces, by different mechanisms, a consistent reduction of GHF (82).

In summary, a complex variety of mechanisms leading to the damage of arteries in CKD patients, generating stiffness in the aorta and central arteries and increased PP and CAP. Additionally, a higher central pulsatility is transmitted into the microcirculation of various tissues, including kidneys, favoring and accelerating its deterioration. A better knowledge of these pathways and processes leading to this vicious circle of the two-way path between arterial stiffness and renal dysfunction will give the medical community better possibilities to improve preventive and therapeutic strategies to reduce vascular injuries and CKD progression, and finally, cardiovascular events.

## Author Contributions

FI, PF, and CC conceived of the presented idea. AC wrote the paper with input from the other authors. All the authors reviewed the final version of the manuscript.

## Conflict of Interest

The authors declare that the research was conducted in the absence of any commercial or financial relationships that could be construed as a potential conflict of interest.

## Publisher's Note

All claims expressed in this article are solely those of the authors and do not necessarily represent those of their affiliated organizations, or those of the publisher, the editors and the reviewers. Any product that may be evaluated in this article, or claim that may be made by its manufacturer, is not guaranteed or endorsed by the publisher.
